# Association between serum type-I, -III and -IV collagen concentrations and inguinal hernia status in piglets

**DOI:** 10.2478/jvetres-2026-0033

**Published:** 2026-06-30

**Authors:** Adam Brodzki, Janusz Kocki, Paulina Gawin, Rafał Maszkowski, Magdalena Krakowiak

**Affiliations:** 1 Department and Clinic of Animal Surgery, Faculty of Veterinary Medicine, University of Life Sciences in Lublin, 20-950 Lublin, Poland; 2 Department of Clinical Genetics, University of Medicine in Lublin, 20-080 Lublin, Poland

**Keywords:** collagen types I, III and IV, connective tissue, ELISA, inguinal hernia, piglets

## Abstract

**Introduction:**

Inguinal hernias are a significant health and economic problem in pig farming, affecting animal welfare and production profitability. Suspected aetiological factors include disorders in the metabolism of extracellular matrix components, including collagen, which is responsible for the integrity and strength of connective tissue. The aim of the study was to compare the concentrations of type-I, -III and -IV collagen in the blood serum of pigs with inguinal hernias to those in healthy animals.

**Material and Methods:**

The study used 87 pigs, comprised of 59 hernia-affected individuals in the experimental group and 28 unaffected counterparts in the control group. The animals were of the same age and came from the same genetic line. The study used ELISA tests for the quantitative measurement of serum collagen concentrations.

**Results:**

The results showed significantly higher concentrations of type-I (P-value < 0.001) and type-III (P-value < 0.01) collagen in the experimental group, indicating an association between collagen metabolism and hernia status. For type-IV collagen, the differences were not statistically significant.

**Conclusion:**

Changes in type-I and type-III collagen levels may be associated with inguinal hernia status in pigs and warrant further investigation as potential risk biomarkers. Type-IV collagen showed no relationship with hernia status in the study population.

## Introduction

Hernias are among the most common conditions encountered in general surgery in both humans and animals, including pigs. Their occurrence constitutes a significant problem on both large and small farms. Among pig congenital defects, hernias are some of the most frequently reported anatomical abnormalities. In contrast to many others, inguinal hernias are additionally associated with a high risk of complications and often necessitate special surgical intervention or the culling of affected animals, presenting a substantial challenge for veterinarians and causing loss to livestock producers. When hernias affect some pigs in the herd, it detracts from animal welfare and entails increased production costs. Animals with hernias show poorer gains in an increase in feed conversion ratio. Not performing surgery may lead to complications including death and is a partial cause of a lower meatiness class for a hernia-suffering pig’s carcass. Animals with hernias remain in a state of compromised welfare, adding to the cumulative economic burden associated with their management. The need for surgical intervention, potential culling, reduced feed efficiency and compromised welfare collectively contribute to the economic burden of inguinal hernia, thereby reducing the profitability of pig farming. Comparable and standardised epidemiological data enabling the assessment of regional or international differences in swine hernia prevalence are lacking, highlighting a significant gap in the veterinary literature and suggesting a research area to inform farm strategy for hernia management.

The formation of hernias is affected by both genetic and environmental factors. Umbilical hernias are estimated to affect 0.4%–1.2% of the pig population (5, 22, 27), with an estimated heritability of 0.25 (29). Scrotal hernias occur at a frequency ranging from 0.5 to 1.5%, with an estimated heritability of 0.3 in different breeds (5, 18, 31), left-sided instances being more common (19). Inguinal hernia affects both sexes. In male pigs with suspected hernias, only a clinical examination allows an inguinal occurrence to be distinguished from a scrotal one. The aetiology of the development of inguinal and scrotal hernias is still unclear; some authors have suggested that two of the causes are abnormal obliteration of the vaginal process and abnormal involution of the internal inguinal ring as failure to close properly, both occurring after the testicles have descended into the scrotum (1, 11). Other authors have proposed causation by an abnormally wide inguinal canal resulting from its non-physiological stretching by the swelling gubernaculum or by hyaluronan accumulation or impaired degradation of hyaluronan. These are factors that predispose a piglet to the development of inguinal hernia during or after the descent of the testicles (1). Hernias may be favoured by impaired innervation of the groin region (23) or low mechanical strength of tissue in the area (2), resulting from metabolic disorders and hydrolysis of collagen and muscle fibre structures (3), which both impair connective tissue repair (7). Each hernia comprises three components: the hernial ring, defined as a defect in the abdominal wall the location of which determines the type of hernia; the hernial sac formed by the peritoneum; and the hernial contents, typically consisting of intestinal loops or omentum (17).

Collagen is the most abundant fibrous structural protein in animals. It forms a right-handed helix that is comprised of three parallel, left-handed, similar or different polypeptide chains. It is also the most abundant component of the extracellular matrix (ECM) (30) and its main wound-healing component. The most common collagen is type I (9), and type III is the second major material in most ECMs. Type V collagen is essential for the formation of type I collagen fibres and determines the final quality of the tissue (20).

Type I collagen is a mature form of collagen and the most stable. Type III collagen represents an immature isoform that is found at higher concentrations in the ECM of patients with and inguinal and incisional hernias (4, 32). According to Bendavid (3), collagen is associated with the pathogenesis of human inguinal hernias. This association appears to be mediated, at least in part, by alterations in collagen composition, whereby an increased type-III collagen content and a reduced type-I:type-III collagen ratio may contribute to structural changes (15).

Understanding the different types of collagen and their properties and roles in connective tissue integrity is important for the diagnosis, treatment and prevention of hernias. Further research in this field may contribute to the development of more effective strategies for preventing the recurrence of inguinal hernias. While advances in biomedical approaches such as gene or cell therapy and biomaterials for the repair and strengthening of the abdominal wall through collagen overproduction and modification have been explored, their application in production animal medicine is likely to be limited. However, insights derived from such research may contribute to a better understanding of tissue repair mechanisms and inform future approaches to herd health management.

The available literature reports a large body of research into the role of connective tissue components in physiological processes and in the aetiology of various diseases and defects, including hernias. Therefore, it appears appropriate to determine the relative proportions of connective tissue components in animals suffering from hernia defects. An assessment of the relationships between their serum concentrations would enable both the monitoring of sick animals in the herd and the development of good manufacturing practice in pig farming. To this end, a research hypothesis was formulated that in animals suffering from inguinal hernias, the levels of type-I, -III and -IV collagen will differ significantly from those of unaffected animals. If proved, this hypothesis would suggest that disturbances in the metabolism of connective tissue components are involved in the pathogenesis of inguinal hernias. The aim of the study was to assess the concentrations of type-I, -III and -IV collagen in the blood serum of pigs with inguinal hernias, as compared to those in the serum of hernia-free pigs.

## Material and Methods

Samples were collected from a commercial farm in southern Poland housing approximately 16,000 animals in an open production system, including 2,285 sows. In a farrowing batch comprising 102 sows and 1,300–1,500 piglets, the incidence of inguinal hernias was approximately 4–5%. The study had 87 animals as the sample, comprising 28 pigs in a control group and 59 pigs in an inguinal hernia group. All the animals were of the same age, specifically piglets in their third week of life (18–21 days old). All of them originated from sows and boars of the same genetics. Only intact male pigs were included in the study, since inguinal hernias are rarely observed in gilts. This selection enabled the formation of a statistically homogeneous study group and controlled for the potential confounding effect of castration on connective tissue metabolism. From the anterior vena cava of each animal, 7.5 mL of blood was collected using 1.2 mm × 40 mm 18G needles (KDM, Berlin, Germany) and blood collection tubes (KABE Labortechnik, Nümbrecht, Germany) containing granules and a blood clotting accelerator. The serum obtained after centrifugation at 1,700 rpm for 15 min using a TDL-4ZB centrifuge (Hunan XINGKE Scientific Instruments Co., Ltd., Hunan, China) was divided into Eppendorf test tubes and stored at –80°C until testing.

Quantitative *in vitro* measurements of serum biomarkers were performed using commercially available ELISA kits for the determination of type-I, -III and -IV collagen concentrations: Pig Collagen Type I (COL1) ELISA Kit, Pig Collagen Type III (COL3) ELISA Kit, Pig Collagen Type IV (COL4) ELISA Kit, all supplied by Abbexa Ltd. (Cambridge, United Kingdom). The ELISA method was chosen for its sensitivity and precision, which allowed the authors to detect low serum collagen concentrations and monitor changes in collagen levels. This method also enables the determination of the exact amount of a studied substance in the blood, and, because of its specificity, enables the identification of a specific type of pig collagen. The tests were conducted in accordance with the manufactuier’s/manufactmers’ protocols and recommendations. Prior to use, the kit components and the samples were brought to room temperature (18–25°C).

For statistical analysis of the results obtained, Statistica 13.3 software (TIBCO Software, Palo Alto, CA, USA) was used. The analysed variables were serum concentrations of type I collagen (COL1), type III collagen (COL3), and type IV collagen (COL4), measured in two groups: a control group of clinically healthy pigs and a study group of pigs diagnosed with inguinal hernia. The Mann–Whitney U test was used to compare differences between groups. Logistic regression analysis and receiver operating characteristic (ROC) curve analysis were performed to evaluate the predictive and diagnostic value of the studied collagen parameters for the occurrence of inguinal hernia. A P-value < 0.05 was considered statistically significant.

## Results

### Collagen of type I

Higher concentrations of type-I collagen in the pig blood serum were noted in the inguinal hernia group than in the control group. The mean concentration in the hernia group was 39.94 ng/mL, whereas in the control group it was 22.79 ng/mL. The median concentration in the group with the defect was 40.86 ng/mL, whereas in the control group, it was barely more than half of this at 21.66 ng/mL. The inguinal hernia group’s results varied more than those of the control group, as evidenced by the higher standard deviation and greater interquartile range (Table 1).

**Table 1.** Type-I collagen concentrations (ng/mL) in 18–21-day-old piglets with and without inguinal hernias

	Inguinal hernia group	Control group
Number	59	28
Mean value	39.94	22.79
Median	40.86	21.66
Minimum	9.652	4.540
Maximum	116.60	56.70
Lower quartile	24.34	12.774
Upper quartile	52.32	28.71
Interquartile range	27.98	15.94
SD	19.03	14.70

A significant difference in type-I collagen concentrations was observed between the inguinal hernia group (n = 59) and the control group (n = 28) (Mann–Whitney U = 388, P-value < 0.001; exact P-value = 0.000042). Higher concentrations were associated with increased odds of belonging to the hernia group (odds ratio (OR) = 1.07 per ng/mL increase, 95% CI: 1.03–1.11; P-value < 0.001).

A classifier based on this concentration exhibited good discriminative performance, with a test power of 0.765 (95% CI: 0.653–0.877; AUC > 0.5) and high statistical significance (P-value < 0.001). A cut-off point of 33.858 ng/mL was identified. Its sensitivity was determined to be 0.61, indicating that 61.0% of pigs with hernias were correctly identified. Specificity was 0.857, showing that 85.7% of healthy animals were correctly classified relative to the identified threshold. Accuracy was 0.690, reflecting that the diagnosis was reliable in 69% of tested pigs. The positive predictive value of 0.9 indicated that 90.0% of pigs with concentrations above the cut-off truly had a hernia, and the negative predictive value of 0.511 indicated that 51.1% of pigs with concentrations below the cut-off could be confidently considered healthy. The positive likelihood ratio was 4.271, suggesting that concentrations above the threshold were more common in pigs with hernias than in healthy pigs. The negative likelihood ratio was 0.455, indicating that the probability of a concentration below the cut-off in affected pigs was 45.5% of that in unaffected ones.

### Collagen of type III

The type-III collagen concentrations in pig serum were higher in the inguinal hernia group than in the control group. The mean concentration in the group with the defect was 4.42 ng/mL, whereas it was 2.61 ng/mL in the control group. The median concentration in the inguinal hernia group was 2.83 ng/mL, whereas in the control group it was 2.05 ng/mL. As in the case of type-I collagen, greater variability in results was observed in the inguinal hernia group, as indicated by the higher standard deviation and wider interquartile range compared to the control group (Table 2).

**Table 2.** Type-III collagen concentrations (ng/mL) in 18–21-day-old piglets with and without inguinal hernias

	Inguinal hernia group	Control group
Number	59	28
Mean	4.42	2.61
Median	2.83	2.05
Minimum	0.88	0.75
Maximum	22.10	9.60
Lower quartile	2.09	1.64
Upper quartile	4.99	2.61
Interquartile range	2.90	0.97
SD	4.23	1.99

The Mann–Whitney test with a continuity correction showed a statistically significant difference in type-III collagen concentrations between the inguinal hernia group and the control group (P-value < 0.01). The test power was 0.688 (AUC > 0.5), with statistical significance (P-value < 0.01). A cut-off point of 2.46 ng/mL was identified.

Analysis of the diagnostic performance of type III collagen concentration revealed a sensitivity of 0.712, indicating that 71.2% of pigs with hernia were correctly identified. Specificity was 0.714, showing that 71.4% of healthy pigs were correctly classified relative to the identified threshold. Accuracy was 0.713, reflecting that the diagnosis was reliable in 71.3% of tested pigs. The positive predictive value of 0.84 indicated that 84.0% of pigs with concentrations above the cut-off truly had a hernia, whereas the negative predictive value of 0.541 indicated that 54.1% of pigs with concentrations below the cut-off could be confidently considered healthy. The positive likelihood ratio was 2.492, suggesting that concentrations above the threshold were more common in pigs with hernia than in healthy pigs. The negative likelihood ratio was 0.403, indicating that the probability of a concentration below the cut-off in affected pigs was 40.3% of that in healthy animals.

### Collagen of type IV

Type-IV collagen concentrations in the pig blood serum were higher in the inguinal hernia group than in the control group, as confirmed by both the higher mean value (7.08 ng/mL compared to 5.40 ng/mL) and the median (5.14 ng/mL compared to 3.96 ng/mL). The hernia group was also characterised by greater variability in results, as evidenced by the greater range of values (0.00–26.4 ng/mL) and higher SD of 5.43 ng/mL in relation to 3.75 ng/mL in the control group (Table 3). No statistically significant difference was observed in type-IV collagen concentrations between the inguinal hernia group and the control group (Mann–Whitney test, P-value > 0.05). Logistic regression analysis indicated no associated between type-IV collagen concentration and hernia status. The analysis indicated the relatively low test power (0.588) of this parameter, which suggests its limited usefulness for distinguishing between pigs with hernias and those without.

**Table 3.** Type-III collagen concentrations (ng/mL) in 18–21-day-old piglets with and without inguinal hernias

	Inguinal hernia group	Control group
Number	59	28
Mean	7.08	5.40
Median	5.14	3.96
Minimum	0.00	0.42
Maximum	26.40	16.90
Lower quartile	3.75	2.57
Upper quartile	9.17	8.34
Interquartile range	5.42	5.76
SD	5.43	3.75

## Discussion

Changes in collagen composition may be a cause of hernia development and may have a systemic character. An example is abdominal-wall hernias in humans, which are likely associated with increased collagen degradation in both fasciae and skin observed in skin biopsy studies (13). The occurrence and recurrence of abdominal wall hernias are more frequent in patients with connective tissue disorders (10) and abdominal aortic aneurysms (12), potentially assigning a role to impaired connective tissue metabolism in the pathogenesis of these conditions. Abnormal collagen metabolism may contribute to reduced tensile strength and decreased mechanical stability of connective and scar tissue (6, 8, 24). These deficits may be associated with altered ratios of one collagen type to others, which may result from both modifications in their synthesis and imbalances in their degradation.

The integrity of the abdominal wall is closely associated with preserved muscular and fascial structures; the mechanical properties of the fascia largely depend on the type of connective tissue and its components, particularly collagens. Type-I collagen, a strong and widely distributed collagen in the human body, is present in structures such as fascia, skin, ligaments and fibrous tissues, and is responsible for the mechanical resistance of tissues. Type-III collagen, abundant in rapidly regenerating tissues such as skin and blood vessels, is more elastic than type I collagen, with which it often coexists within the same fibres (6, 28). In the skin, the dominant form is mature, mechanically stable type-I collagen, but in skin in the early stages of wound healing, immature and mechanically less stable type-III collagen is often observed (26). Alterations in the ratio of type-I to type-III collagen are detectable at the mRNA level (24, 25, 34) and have been observed in the skin (32) and fascia (21) of patients with hernia, suggesting a systemic nature of these defects. Changes in the relative proportions of these two collagen types may affect tissue elasticity and strength and foster hernia formation. These changes may be sequelae of metabolic disturbances, which were suspected in patients with hernias exhibiting an increased amount of type III collagen (15).

Literature reports support these observations. In studies by Klinge *et al*. (15, 16), both immunohistochemical analysis and Western blotting demonstrated a significant decrease in the type-I-to- type-III collagen ratio in the skin of patients with indirect or direct inguinal hernias, accompanied by an increase in type-III collagen levels. Zhao *et al*. (33) reported elevated type-III collagen expression in patients with parastomal hernia, along with a significantly decreased type-I:-III collagen ratio, suggesting a key role of type-III collagen in parastomal hernia pathogenesis.

Our own findings are consistent with these observations and support an association between altered collagen metabolism and inguinal hernia status in pigs. Significant differences in type-I collagen levels were observed between pigs with inguinal hernia and healthy controls. The median collagen concentration in the affected pig group was nearly double that of the unaffected pig group, with statistical significance at P-value < 0.00. Logistic regression analysis also indicated an association between higher type-I collagen levels and hernia status, with each unit increase in type I-collagen concentration (ng/mL) corresponding to approximately a 7% increase in likelihood of belonging to the hernia group. The ROC curve analysis and AUC confirmed that type-I collagen concentrations effectively discriminated between pigs with hernia and those without, demonstrating good diagnostic performance for this parameter.

With respect to type-III collagen, our study revealed a statistically significant difference in its concentrations between the hernia group and the control group (P-value < 0.01). However, logistic regression analysis did not show a statistically significant association between increased type-III collagen levels and hernia status. The ROC curve analysis nevertheless can be suggested to have classified pigs with hernia when the type-III collagen concentration exceeded the threshold. These findings indicate a complex relationship between type-III collagen levels and hernia occurrence, suggesting that this concentration may serve as an auxiliary marker, whereas the key genetic and diagnostic relevance lies in the disturbed ratio of type-I to type-III collagen. Reports by Henriksen *et al*. (14) indicate a heterogeneous pattern of collagen metabolism in patients with hernias, highlighting significant alterations in type-IV collagen turnover. The authors demonstrated that increased turnover within the basement membrane may be indicative of the presence of inguinal or incisional hernias, and that alterations in collagen metabolism may be a precondition for hernia development. These findings suggest that not only the quantity of collagen but also the dynamics of its metabolism may constitute an important risk factor. The studies also indicate that increased collagen turnover can contribute to matrix destabilisation, thereby promoting hernia formation (14). In our own study, no statistically significant differences in systemic type-IV collagen levels were observed between the hernia and control groups, indicating no direct correlation between its concentration and hernia status. The analyses showed that type-IV collagen did not differ significantly between pigs with hernias and controls, either in terms of serum levels or as a potential predictor of hernia status.

Both the findings of Henriksen *et al*. (14) and our own provide valuable insights into the role of type-IV collagen in hernia development; however, their interpretation must be approached with caution. An important consideration in this context is the difference between local and systemic measurements. Henriksen *et al*. (14) assessed collagen turnover at the level of the basement membrane, whereas the present study evaluated circulating collagen concentrations. Local alterations within the extracellular matrix may not be directly reflected in serum levels, which could account for the lack of association observed in this study. Clarifying the relationship between local ECM changes and systemic markers is important for the interpretation of collagen-based indicators in hernia research. In particular, understanding how local alterations within the ECM may influence systemic metabolic indicators is critical for the development of more effective diagnostic and therapeutic strategies for hernias.

It should be emphasised that the available literature on collagen metabolism disturbances in the context of inguinal hernias pertains almost exclusively to human studies. To date, no analysis has been carried out of systemic levels of type-I, -III and -IV collagen in pigs with inguinal hernias, despite the species being an important model in both veterinary medicine and translational research. In this context, our study fills a gap in the literature by providing the first data on the collagen profile in the serum of pigs with this defect. These results provide a foundation for further investigations into the mechanisms underlying hernia pathogenesis in animals and the potential use of collagen markers in the diagnosis and prevention of this condition.

## Conclusion

Elevated levels of type-I collagen and type-III collagen in the serum of pigs with inguinal hernias indicate their possible involvement in the pathogenesis of this defect. To date, no similar studies have been conducted to assess the collagen levels in the serum of pigs with hernias. The results obtained may provide a basis for further research on both the use of collagen as a biomarker and the molecular mechanisms of the development of hernias in pigs.
